# The Prevalence of and Factors Associated with the Use of Smoking Cessation Medication in Korea: Trend between 2005–2011

**DOI:** 10.1371/journal.pone.0074904

**Published:** 2013-10-09

**Authors:** Dong Wook Shin, Beomseok Suh, Sohyun Chun, Juhee Cho, Sang Ho Yoo, Seok Joong Kim, Bumjo Oh, Belong Cho

**Affiliations:** 1 Department of Family Medicine, Seoul National University Hospital, Seoul, Korea; 2 Center for Health Promotion and Optimal Aging, Seoul National University Hospital, Seoul, Korea; 3 Cancer survivorship clinic, Seoul National University Cancer Hospital, Seoul, Korea; 4 Department of Health Sciences and Technology, Samsung Advanced Institute for Health Sciences and Technology, Sungkyunkwan University School of Medicine, Seoul, Republic of Korea; 5 Department of Epidemiology, Johns Hopkins Bloomberg School of Public Health, Baltimore, Maryland, United States of America; 6 Department of Health, Behavior and Society, Johns Hopkins Bloomberg School of Public Health, Baltimore, Maryland, United States of America; 7 Department of Family Medicine, Hallym University Hospital, Pyeongchon, Korea; 8 National Medical Center, Seoul, Korea; Universidad Europea de Madrid, Spain

## Abstract

**Background:**

In Korea, nicotine replacement therapy (NRT) has been widely used in government-led, public health center-based smoking cessation services since 2004 and varenicline has become available from 2007 but without reimbursement. In this study which used a series of nationwide cross-sectional surveys in Korea performed from 2005 to 2011, we examined the prevalence of smoking cessation medication use and factors associated with it.

**Methods:**

We analyzed data from the third to fifth waves of Korean National Health and Nutrition Examination Survey (2005–2011). Prevalence of each smoking cessation method use was calculated for each year, and its secular trend was tested by multivariate logistic regression.

**Results:**

Among smokers who made quit attempt during the previous year, 15.7% had used smoking cessation medications,15.3% had used NRT, and 0.7% had used prescription medication. There was a significant increasing trend for NRT use (P<0.001) during the study period, but use of prescription medication did not show any increase over time (P = 0.654) Education on smoking prevention and cessation was associated with smoking cessation medications use (OR 2.08, 95% CI 1.58–2.75).

**Conclusions:**

While the use of NRT has increased over years through government-sponsored smoking cessation programs, use of prescription drugs remained very low and flat probably due to lack of reimbursement. Education of smokers about effective smoking cessation methods and change in reimbursement policy are suggested to stimulate evidence-based smoking cessation practice.

## Introduction

Smoking is the major cause of preventable mortality worldwide, accounting for cancer and cardiovascular disease [1]. Faced with the facts, almost half of all smokers attempt to quit smoking each year, but with only 3% success rate [2], [3]. Current guidelines for smoking cessation recommend use of effective medications for tobacco dependence in all smokers, except when medically contraindicated or with specific populations for which there is insufficient evidence of effectiveness (e.g. pregnant women, smokeless tobacco users, light smokers, and adolescents)[4]. In addition, use of smoking cessation medication is used as one of the indicators to evaluate the effectiveness of tobacco cessation interventions from the public health perspectives [5].

Use of smoking cessation medication was studied in western countries, including US [2], UK [6], Switzerland [7], and so on. Those studies showed that many smokers attempt to quit unassisted (“cold turkey”), or opt for treatment with unknown efficacies [8]. Such underuse of efficacious pharmacological treatment for smoking cessation was suggested to at least partly explain the discrepancy between quit intention and success [8].

Treatment utilization may differ by different cultural and health system factors [9]. However, to our knowledge, there has been no study which investigated the prevalence of smoking cessation medication use in Korean smokers trying to quit. In Korea, nicotine replacement therapy (NRT) has been available over-the-counter since 1995. As it does not require prescription by a physician, it has become widely used in government-led, public health center-based smoking cessation services throughout Korea [10], as well as in company and university health promotion programs. Bupropion SR (Wellbutrin, sustained-release) and varenicline (Champix) have been available since 2002 and 2007 by prescription, respectively, but are still not reimbursed.

In this study which used a series of nationwide cross-sectional surveys in Korea performed from 2005 to 2011, we examined the prevalence of smoking cessation medication use and factors associated with it. This would reveal how the public health center-based government smoking cessation programs and introduction of varenicline may have influenced the use of smoking cessation medication in Korea. It will also contribute to understanding the use of smoking cessation medication in Asia.

## Methods

### Data Source and Participants

We analyzed data from the third to fifth waves of Korean National Health and Nutrition Examination Survey (KNHANES), which were conducted in 2005 (third), 2007–2009 (fourth), and 2010–2011 (fifth). The KNHANES is a nationally representative cross-sectional survey on the health and nutritional status of non-institutionalized Korean civilians, periodically conducted by Korean Center for Disease Control and Prevention (KCDC). Sampling units were by households from which data was collected through a stratified, multistage, probability-sampling design based on sex, age, and geographical area using household registries.

The participants for each KNHANES survey were informed that they had been randomly selected as a sample household, and voluntarily participated in the survey. A written informed consent was obtained. Average overall participation rate was around 80% during the study period. Each of the population was assigned a weighted value on the basis of geographical and demographic characteristics to allow estimates to be calculated for the entirety of the Korean population. As this study involved open data without any identifier, ethical review was not needed according to the policy of Seoul National University Hospital institutional review boards.

### Measures

The questionnaires related to smoking were self-reported. All respondents were asked about their current smoking status (by WHO definition) [11], and their daily cigarette consumption amount. Current smokers were subsequently asked whether they had made a serious quit attempt during the past 12 months (“During the past 12 months, have you made any serious attempt to stop smoking” in 2005; “During the past 12 months, have you made any attempt to stop smoking lasting 24 hours or more” in 2007).

Smokers were also asked “Have you used any of the following methods to help you quit smoking?” The smoking cessation methods that respondents were queried about were: (1) NRT, including patch, gum, etc (over-the-counter or prescription was not discriminated); (2) prescribed medications (bupropion SR or varenicline was not discriminated); (3) smoking cessation herb, called “geumyeoncho” in Korea; (4) and others, which included quit line, smoking cessation acupuncture, and smoking education and counseling. Respondents were allowed to choose from more than one method, if they used more than one method. Smokers in the 4^th^ and 5^th^ waves were also asked whether they had participated in any education program about smoking prevention or cessation in the previous year.

Data on sociodemographic characteristics were also collected and included age, sex, education level, working status, household income, and perceived health status (as 1, very good to 5, very poor).

### Statistical Analysis

Primary analysis was limited to current smokers who reported having attempted to quit smoking during the past 12 months. Prevalence of each smoking cessation method use was calculated for each year, and its secular trend was tested by multivariate logistic regression. Use of multiple methods was not considered in the analyses, and each outcome was separately analyzed.

Multivariate logistic regression analyses were used to identify the factors associated with smoking cessation medication use. Age, sex, education level (categorized by high school and above, and less than high school), working status (employed, self-employed vs. unemployed, retired, student, housewife), income status (categorized by upper half, and lower half), and subjective health status (categorized as very good and good, fair, bad and very bad), daily cigarette consumption (categorized as <10, 10 to 19, and ≥20 cigarettes per day), and survey year was included as independent variables. For subsamples of 4^th^ and 5^th^ year, we also included whether they had participated in any education program about smoking prevention or cessation in the previous year.

Weighted values of each subject were used to account for the complex sampling design, according to the KNHANES analytic guide published by KCDC. Statistical analyses were carried out using STATA software (version 12.0; STATA corp., Houston, TX). P-value <0.05 (two sided) was regarded statistically significant.

## Results

### Study Participants

**Figure 1:**
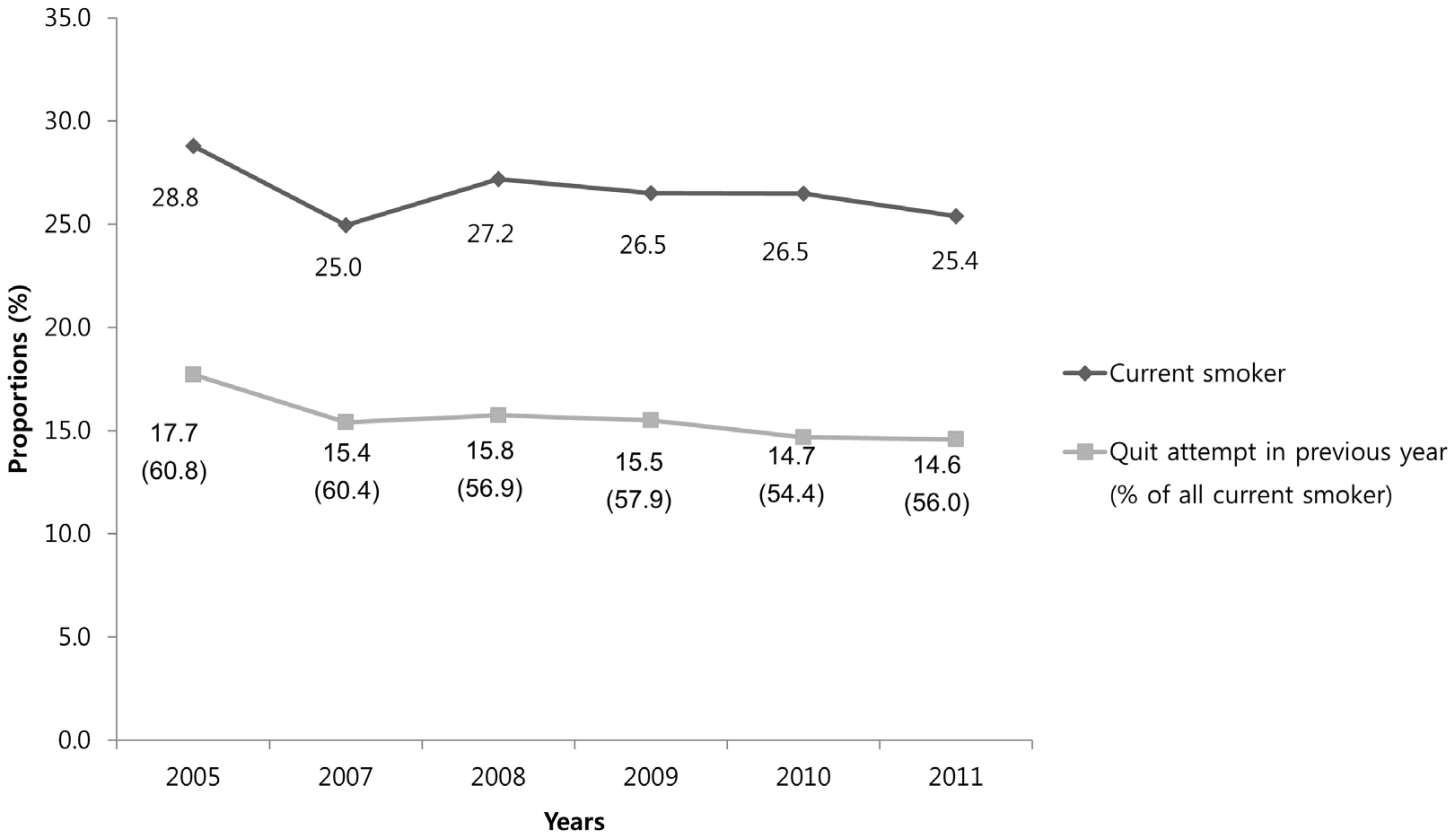
Prevalence of current smoking rate and quit attempt among current smokers.

A total of 37,689 adults aged 19 years or more participated in the health behavior survey of KNHANES during the defined study period. Among them, 8382 (26.4%, weighted) were current smokers. Of them, 4,788 (57.7%, weighted) who reported having had any serious quit attempt in the previous year were included in primary analyses. There was no significant trend in the current smoking rate (P for trend = 0.139), but there was a slightly decreasing trend of quit attempts (P for trend = 0.002) **(** **Figure 1** **)**.

Mean age (standard error) of the participants was 40.2 (0.3), and most were male (87.9%). Most had at least high school education (79.1%), and were working (77.7%). Average daily cigarette consumption was 15.5 **(** **Table 1** **)**.

**Table 1:** Characteristics of study subjects (N = 4,788).

	Total sample (N = 4,788)		Subsample (N = 3,570)	
	Unweighted N	Weighted proportion (%)	Unweighted N	Weighted proportion (%)
Age, mean (SE)		40.2 (0.3)		40.5 (0.3)
Sex				
Male	4104	87.9	3024	87.4
Female	684	12.1	546	12.6
Education				
Less than high school (<12 years)	1263	20.9	999	21.7
High school and above (≥12 years)	3511	79.1	2557	78.3
Employment status				
Working	3634	77.7	861	77.9
Not working	1137	22.3	2692	22.1
Income				
Lower half	2505	50.8	1817	49.6
Upper half	2213	49.2	1701	50.4
Self-reported health status				
Very good, good	1825	37.6	1300	36.0
Fair	2025	45.2	1549	46.5
Poor, very poor	929	17.2	712	17.5
Daily cigarette amount				
0–9	1174	23.3	876	23.3
10–19	1885	40.9	1419	41.0
≥20	1727	35.8	1274	35.7
Round				
III (2005)	1218	18.5		
IV (2007–2009)	2137	49.5	2137	60.8
V (2010–2011)	1433	32.0	1433	39.2

N: number.

**Figure 2:**
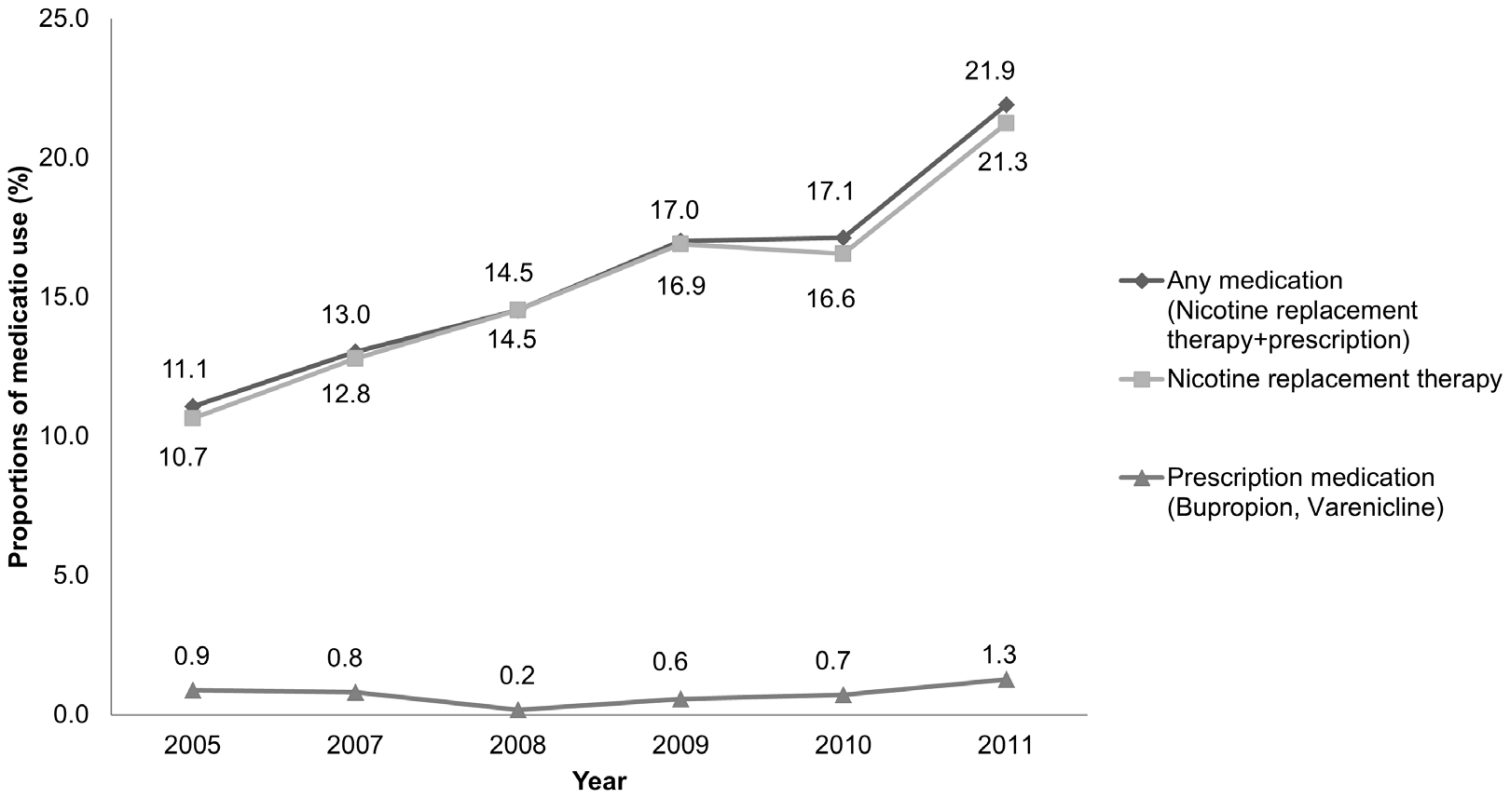
Prevalence of smoking cessation medication use during 2005–2011.

Among smokers who made quit attempt during the previous year, 15.7% had used smoking cessation medications, 15.3% had used NRT, and 0.7% had used prescription medication. There was a significant increasing trend for NRT use (P<0.001) during the study period, but use of prescription medication did not show any increase over time (P = 0.654) **(** **Figure 2** **)**. Use of smoking cessation herb was reported in 8.5% of those who made any attempt to quit, and remained stable throughout the period (P = 0.844).

Table 2 shows the odds ratio (OR) and 95% confidence interval (CI) of factors associated with the use of smoking cessation medication in current smokers who had made any quit attempt during the previous year. The use of pharmacologic medications was significantly higher in smokers who smoked 10–19 cigarettes per day (adjusted odds ratio [aOR] 1.88, 95% confidence interval [CI] 1.38–2.54) and ≥20 cigarettes per day (aOR 2.69, 95% CI 1.96–3.69, respectively) than smokers who smoked <10 cigarettes per day. Higher income was associated with higher use (aOR 1.22, 95% CI 1.01–1.48). There was an increasing trend by survey year (aOR 1.15, 95% CI 1.09–1.20). In subgroup analysis, education on smoking prevention and cessation was associated with smoking cessation medications use (OR 2.08, 95% CI 1.58–2.75). There were no associations in age, sex, household income, working status, and perceived health status with smoking cessation medication use.

**Table 2:** Factors associated with smoking cessation medication use.

	Total sample (KNHANES III–V)				Subsample (KNHANES IV–V)		
	weighted proportion (%)	Univariate OR (95% CI)	Multivariate OR (95% CI)		weighted proportion (%)	Univariate OR(95% CI)	Multivariate OR(95% CI)
Year (per year)			1.15 (1.09–1.20)				1.17 (1.08–1.28)
Age (per year)			1.00 (0.99–1.01)				1.00 (0.99–1.01)
Sex							
male	16.1				17.2		
female	12.6	0.75 (0.56–1.01)	1.03 (0.74–1.43)		13.1	0.72 (0.52–1.00)	1.05 (0.72–1.51)
Education							
Less than high school	16.7				17		
High school and above	15.4	0.91 (0.72–1.15)	0.98 (0.73–1.31)		16.6	0.97 (0.74–1.26)	1.01 (0.72–1.40)
Working							
Not working	14.5				16.3		
Working	16	1.12 (0.89–1.42)	0.91 (0.71–1.18)		16.8	1.03 (0.80–1.35)	0.81 (0.61–1.08)
Income status							
Lower half	14.5				15.7		
Upper half	16.6	1.18 (0.99–1.42)	1.22 (1.01–1.48)		17.6	1.15 (0.94–1.41)	1.17 (0.94–1.46)
Health status							
Very good∼good	14.2				15.6		
Fair	16.4	1.18 (0.96–1.46)	1.08 (0.87–1.33)		17.3	1.13 (0.90–1.42)	1.06 (0.84–1.35)
Very bad ∼bad	17.0	1.24 (0.95–1.61)	1.14 (0.85–1.53)		17.3	1.13 (0.84–1.53)	1.07 (0.77–1.48)
Smoking amount							
<10 per/day	9.0				9.7		
10–19 cigarettes/day	15.3	1.82 (1.36–2.44)	1.88 (1.38–2.54)		16.6	1.85 (1.34–2.56)	1.88 (1.34–2.65)
≥20 cigarettes/day	20.5	2.60 (1.94–3.48)	2.69 (1.96–3.69)		21.5	2.55 (1.84–3.54)	2.71 (1.89–3.88)
Education about smoking prevention or cessation in previous year							
No	NA			15.0			
Yes	NA		NA	26.6		2.05 (1.57–2.68)	2.08 (1.58–2.75)

OR: odds ratio; CI: confidence interval.

All variables in the univariate analyses were included in multivariate model.

## Discussion

To our knowledge, this is the first report about the prevalence and factors of smoking cessation medication use in a non-western country. Strengths include use of nationally representative data, and investigation of secular trends.

Overall, only 15.7% of smokers who had made any quit attempt during the past 12 months reported having used smoking cessation medication, and most of them used NRT. While studies persistently reported underuse of smoking cessation medication in quit attempts, figures in our study is even lower than those reported in previous studies: The reported prevalence of smoking cessation medication was 48.4% in UK (as of 2007) [6], 32.2% in US (as of 2003) [2], and 24% in Switzerland (as of 2008–2009) [7].

Smokers’ attitudes toward smoking cessation medication largely determine the decision to use it. Skepticism about the effectiveness and concerns about the safety of smoking cessation medication was reported as a reason for underuse of smoking cessation medication [8], [12]. Previous studies from Switzerland and US showed that only 1 or 2 of 6 participants agreed with the effectiveness of NRT for smoking cessation [7], [8], and many smokers underestimated the effectiveness of smoking cessation medication [12]. In addition, two-thirds of all US respondents harbored concerns about the safety of NRT, believing that “NRT was as harmful as cigarettes” [8] and “nicotine can cause a heart attack or cancer” [13]. Swiss smokers also had concerns that NRT may maintain dependence by developing a new dependence [7]. The term ‘nicotine’ is often used as a code word for the entire tobacco problem [14], creating association of nicotine and harms of smoking. Long and detailed warning message from FDA for the NRT, compared to short warning message of cigarette packaging, may also have contributed to smokers’ reluctance to use the products [7], [14]. Although previous data mainly addressed specifically misperceptions regarding NRT, similar misperceptions may also be attached to other medications and treatments [8]. While few data are available regarding smokers’ perception of bupropion SR and varenicline, publicity on the recent debate regarding potential risk of suicide [15], and cardiovascular events [16], [17] may discourage smokers not to seek assistance in their attempt to quit smoking.

Another important factor of low use may include simple ignorance about the existence of smoking cessation medication [12]. In a survey on Swiss smokers regarding NRT, one fifth of the smokers answered that they neither know nor used the NRT [7]. In a US study where almost all study respondents reported they have heard of nicotine patch and gum, only small proportions of them had heard about bupropion SR (63%), nicotine inhaler (41%), and nicotine spray (9%) [13]. Promoting the availability of evidence-based cessation methods may be suggested to improve the usage of efficacious cessation aids [18].

Cultural norms affect treatment-seeking behaviors related to smoking cessation [9], and may partly account for lower use in Korea. US studies showed that ethnicity could be a factor that can influence the utilization of treatment [9], [19], and non-white respondents were more likely than white respondents to harbor concerns about safety and efficacy of smoking cessation medication [8]. It is also recognized that Asian Americans with addictive disorders face several cultural and practical barriers to treatment and the result has been an underutilization of addiction and mental health treatment. For example, shame in asking for help for an addictive disorder is perceived as one of the most recognized cultural barrier in this population [20]. There are some preliminary evidence for the effectiveness of such culturally appropriate smoking cessation intervention [21].

Our study shows that education on smoking prevention and cessation doubles the usage of smoking cessation medications. This may be because the education made smokers become aware of the availability of effective smoking cessation medication and to have better knowledge of and a more positive attitude toward medication. It is also possible that the NRT trial or referral to smoking cessation services were provided after the education session, which is currently a common practice in Korea [22], [23]. As attitudes toward medication was more positive in ever-users than in never-users [7], offering reluctant smokers an opportunity to try smoking cessation medication on a trial basis may be a way to improve knowledge about the safety and efficacy of these medications, and eventually increase smoking cessation [13].

The by far most commonly used medication was NRT. Furthermore, there was a significant trend in the overall use of NRT during the study period. This may be attributable to government-sponsored programs which were launched in 2004 under the direction of the Ministry of Health and Welfare. In this nationwide program, 253 public health centers located in all administrative districts provide free behavioral counseling and medication (mostly NRT) [10]. Such increase in use of NRT was also experienced in UK (12% in 2000 to 28% in 2006), where a government strategy to systematically encourage treatment use was implemented by promoting both medications and specialist behavioral treatment [6]. Therefore, implementing government-led strategies can be considered effective to promote smoking cessation medication use.

In contrast, usage of prescription medication was very low and flat throughout the study period, even with the advent of an effective smoking cessation drug, i.e. varenicline. This is in contrast with the UK situation, where the use of varenicline increased from 0% in 2006 to 4.3% in 2008 resulting in decrease in the use of OTC NRT during the same period [6]. Such difference may be due to the difference in reimbursement policy. While physician’s prescription for smoking cessation are reimbursed in the NHS-based UK primary care system [6], both prescription for smoking cessation and the drugs themselves are totally based on out-of-pocket cost in a fee-for-service based primary care system in Korea. A survey on Korean physicians with experience in smoking cessation services identified that high cost of medication for smoking cessation due to non-reimbursement (50.7%) and the absence of a medical fee on smoking cessation services (34.8%) were main barriers to active involvement in smoking cessation services [24]. Therefore, change in the primary care reimbursement policy would be the necessary condition to maximize the use of prescription medication.

Heavy smokers were more likely to use smoking cessation medication. This is consistent with previous research, which also suggested that more heavily addicted smokers are more likely to use assistance in quitting [2], [15], [25], [26]. Patients’ own perception of high nicotine dependence, as well as physician’s propensity to recommend medication to heavy smokers, may explain the result. Higher income was weakly, but significantly associated with higher smoking cessation medication use, suggesting disparity in access to the appropriate services. As NRT and counseling is provided for free in public health centers, it is likely that people with lower income do not have enough time for visiting public health centers. No association between the use of smoking cessation medication and other sociodemographic characteristics, such as age, sex, and educational status was observed.

Despite of the increase in smoking cessation medication use, we did not observe decline of overall smoking rate during the observation period, implying that most of the smokers’ attempts were unsuccessful. There are some criticism about emphasis on smoking cessation medication and advocacy for unassisted cessation [27]. Critics concern about the increasing medicalization and commodification of cessation. They also argue that there is no population-based evidence of smoking rate decline with the use of smoking cessation medication [28], [29], probably due to the lower effectiveness of the medication in the ‘real world setting’. However, as shown in our study and others, more dependent smokers gravitate towards treatment [2], [15], [25], [26], and such self-select would lead to a bias toward null [30]. In addition, some observation studies also suggest improved quit rates among smokers who made self-initiated quit attempts and used medication [31], [32]. Even if the use of smoking cessation medication can increase the chance of success in individuals, limited utilization itself can make it difficult to detect any public health impact in the population level. Furthermore, staggering of smoking prevalence in Korea since 2007 is mainly due to weak tobacco control policies in Korea [33].

In addition, the way smoking cessation medication is used is often not optimal. Under dose, and early discontinuation are common and reduce the effect of medication [30], [34]. Furthermore, only 1/5 of the smoking cessation medication users had adjuvant behavioral counseling [2], [35]. Therefore, even faced with absence of decline in smoking cessation rate with increase in smoking cessation medication, we assert that the more appropriate approach should be encouraging the use of such medication with concomitant behavioral counseling to raise the effectiveness [26]. Reinforcing the smokers’ motivation and optimizing treatment dosage strengths, formulation, and duration may result in a more pronounced effect on population trends in smoking behavior [26]. Depriving the smokers with nicotine dependence of the intervention with proven efficacy at least in randomized clinical trial settings will not solve the problem, and could be even viewed as unethical. No treatment can help a smoker who does not use it. Therefore, increasing utilization of effective treatment along with good behavioral support would be an appropriate approach to decrease smoking rates in the general population.

There are several limitations to be mentioned. First, this study relies on self-reported data, leading to recall bias and probably overestimation of smoking cessation medication use. Second, as this study is a secondary analysis of KNHANES, which the authors did not develop for the study purpose, the study lacked detailed measures about the perceived efficacy and safety concerns, and their experiences. A valid and reliable measure of such knowledge and attitudes would have enhanced the understanding of the reasons for underuse of smoking cessation medication [7], [8]. Third, secular trend analysis were performed within relatively short timeframe. However, our data was sufficient to see the effect of the government smoking cessation program, which was launched in 2004, and availability of varenicline, which has been released in the market from 2007.

Despite the above mentioned limitations, our study has important policy implications in public health perspectives for countries, in particular those with similar cultural background and health care system. Although over half of all smokers try to quit each year in Korea, less than one fifths of those who try to quit do so with the benefit of smoking cessation medication with proven efficacy. While the use of NRT has increased over years through government-sponsored smoking cessation programs, use of prescription drugs remained very low and flat probably due to lack of reimbursement. Education of smokers about effective smoking cessation methods and change in reimbursement policy are suggested to stimulate evidence-based smoking cessation practice.

## References

[1] U.S. Department of Health and Human Services (2010) How Tobacco Smoke Causes Disease: The Biology and Behavioral Basis for Smoking-Attributable Disease: A Report of the Surgeon General. Atlanta, GA: U.S. Department of Health and Human Services, Centers for Disease Control and Prevention, National Center for Chronic Disease Prevention and Health Promotion, Office on Smoking and Health. 21452462

[2] Shiffman S, Brockwell SE, Pillitteri JL, Gitchell JG (2008) Use of smoking-cessation treatments in the United States. Am J Prev Med 34: 102–111. 18201639 10.1016/j.amepre.2007.09.033

[3] Hughes JR, Keely J, Naud S (2004) Shape of the relapse curve and long-term abstinence among untreated smokers. Addiction 99: 29–38. 14678060 10.1111/j.1360-0443.2004.00540.x

[4] Clinical Practice Guideline Treating Tobacco Use and Dependence 2008 Update Panel L, and Staff (2008) A clinical practice guideline for treating tobacco use and dependence: 2008 update. A U.S. Public Health Service report. Am J Prev Med 35: 158–176. 18617085 10.1016/j.amepre.2008.04.009 PMC4465757

[5] International Agency for Research on Cancer (2008) IARC Handbooks of Cancer Prevention, Tobacco Control, Vol. 12: Methods for Evaluating Tobacco Control Policies. Lyon, France.

[6] Kotz D, Fidler J, West R (2009) Factors associated with the use of aids to cessation in English smokers. Addiction 104: 1403–1410. 19549267 10.1111/j.1360-0443.2009.02639.x

[7] Etter JF, Perneger TV (2001) Attitudes toward nicotine replacement therapy in smokers and ex-smokers in the general public. Clin Pharmacol Ther 69: 175–183. 11240982 10.1067/mcp.2001.113722

[8] Shiffman S, Ferguson SG, Rohay J, Gitchell JG (2008) Perceived safety and efficacy of nicotine replacement therapies among US smokers and ex-smokers: relationship with use and compliance. Addiction 103: 1371–1378. 18855827 10.1111/j.1360-0443.2008.02268.x

[9] Shiffman S, Brockwell SE, Pillitteri JL, Gitchell JG (2008) Individual differences in adoption of treatment for smoking cessation: demographic and smoking history characteristics. Drug Alcohol Depend 93: 121–131. 17996399 10.1016/j.drugalcdep.2007.09.005

[10] Oh JK, Lim MK, Yun EH, Shin SH, Park EY, et al (2012) Cost and effectiveness of the nationwide government-supported Smoking Cessation Clinics in the Republic of Korea. Tob control 22(e1): e73–77. 22752272 10.1136/tobaccocontrol-2011-050110

[11] World Health Organization (1998) Guidelines for controlling and monitoring the tobacco epidemic. Geneva: World Health Organization.

[12] Hammond D, McDonald PW, Fong GT, Borland R (2004) Do smokers know how to quit? Knowledge and perceived effectiveness of cessation assistance as predictors of cessation behaviour. Addiction 99: 1042–1048. 15265101 10.1111/j.1360-0443.2004.00754.x

[13] Bansal MA, Cummings KM, Hyland A, Giovino GA (2004) Stop-smoking medications: who uses them, who misuses them, and who is misinformed about them? Nicotine Tob Res 6 Suppl 3S303–310. 15799593 10.1080/14622200412331320707

[14] Shiffman S (2010) Smoking-cessation treatment utilization: The need for a consumer perspective. Am J Prev Med 38: S382–384. 20176311 10.1016/j.amepre.2009.12.004

[15] Moore TJ, Furberg CD, Glenmullen J, Maltsberger JT, Singh S (2011) Suicidal behavior and depression in smoking cessation treatments. PloS one 6: e27016. 22073240 10.1371/journal.pone.0027016 PMC3206890

[16] Prochaska JJ, Hilton JF (2012) Risk of cardiovascular serious adverse events associated with varenicline use for tobacco cessation: systematic review and meta-analysis. BMJ 344: e2856. 22563098 10.1136/bmj.e2856 PMC3344735

[17] Singh S, Loke YK, Spangler JG, Furberg CD (2011) Risk of serious adverse cardiovascular events associated with varenicline: a systematic review and meta-analysis. CMAJ 183: 1359–1366. 21727225 10.1503/cmaj.110218 PMC3168618

[18] Willemsen MC, Wiebing M, van Emst A, Zeeman G (2006) Helping smokers to decide on the use of efficacious smoking cessation methods: a randomized controlled trial of a decision aid. Addiction 101: 441–449. 16499517 10.1111/j.1360-0443.2006.01349.x

[19] Fu SS, Sherman SE, Yano EM, van Ryn M, Lanto AB, et al (2005) Ethnic disparities in the use of nicotine replacement therapy for smoking cessation in an equal access health care system. Am J Health Promot 20: 108–116. 16295702 10.4278/0890-1171-20.2.108

[20] Fong TW, Tsuang J (2007) Asian-americans, addictions, and barriers to treatment. Psychiatry (Edgmont) 4: 51–59. PMC2860518 20428303

[21] Chen MS Jr (2001) The status of tobacco cessation research for Asian Americans and Pacific Islanders. Asian Am Pac Isl J Health 9: 61–65. 11720415

[22] Korea Health Promotion Foundation (2011) An Introduction to 2011 Public Health Center Health Promotion Program. Korea Health Promotion Foundation. Seoul, Republic of Korea: Korea Health Promotion Foundation.

[23] Webiste of Korean quit line. http://www.nosmokeguide.or.kr/. Accessed 19 June 2013.

[24] Kim C-H, Song H-R, Lee W-S, Kim J-Y (2009) Attitudes toward Smoking Cessation Intervention and Services among Korean Physicians: A Questionnaire Survey. Korean J Fam Med 30: 857–863.

[25] Hung WT, Dunlop SM, Perez D, Cotter T (2011) Use and perceived helpfulness of smoking cessation methods: results from a population survey of recent quitters. BMC Public Health 11: 592. 21791111 10.1186/1471-2458-11-592 PMC3160379

[26] Cummings KM, Hyland A (2005) Impact of nicotine replacement therapy on smoking behavior. Ann Rev Public Health 26: 583–599. 15760302 10.1146/annurev.publhealth.26.021304.144501

[27] Chapman S, MacKenzie R (2010) The global research neglect of unassisted smoking cessation: causes and consequences. PLoS Med 7: e1000216. 20161722 10.1371/journal.pmed.1000216 PMC2817714

[28] Wakefield MA, Durkin S, Spittal MJ, Siahpush M, Scollo M, et al (2008) Impact of tobacco control policies and mass media campaigns on monthly adult smoking prevalence. Am J Public Health 98: 1443–1450. 18556601 10.2105/AJPH.2007.128991 PMC2446442

[29] Cummings KM, Hyland A, Giovino GA, Hastrup JL, Bauer JE, et al (2004) Are smokers adequately informed about the health risks of smoking and medicinal nicotine? Nicotine Tob Res 6 Suppl 3S333–340. 15799596 10.1080/14622200412331320734

[30] Shiffman S, Rolf CN, Hellebusch SJ, Gorsline J, Gorodetzky CW, et al (2002) Real-world efficacy of prescription and over-the-counter nicotine replacement therapy. Addiction 97: 505–516. 12033652 10.1046/j.1360-0443.2002.00141.x

[31] West R, Zhou X (2007) Is nicotine replacement therapy for smoking cessation effective in the “real world”? Findings from a prospective multinational cohort study. Thorax 62: 998–1002. 17573444 10.1136/thx.2007.078758 PMC2117127

[32] Miller N, Frieden TR, Liu SY, Matte TD, Mostashari F, et al (2005) Effectiveness of a large-scale distribution programme of free nicotine patches: a prospective evaluation. Lancet 365: 1849–1854. 15924980 10.1016/S0140-6736(05)66615-9

[33] World Health Organization (2011) WHO report on the global tobacco epidemic, 2011: warning about the dangers of tobacco. Geneva: World Health Organization.

[34] Shiffman S, Hughes JR, Pillitteri JL, Burton SL (2003) Persistent use of nicotine replacement therapy: an analysis of actual purchase patterns in a population based sample. Tob Control 12: 310–316. 12958394 10.1136/tc.12.3.310 PMC1747733

[35] Pierce JP, Gilpin EA (2002) Impact of over-the-counter sales on effectiveness of pharmaceutical aids for smoking cessation. JAMA 288: 1260–1264. 12215133 10.1001/jama.288.10.1260

